# Supplementary material for: The alarmin interleukin-1α triggers secondary degeneration through reactive astrocytes and endothelium after spinal cord injury
Source: Nat Commun. 2022 Oct 2;13:5786. doi: 10.1038/s41467-022-33463-x (PMC9527244; doi:10.1038/s41467-022-33463-x)
Supplement: Supplementary file 1 — Supplementary Information [file 41467_2022_33463_MOESM1_ESM.pdf]

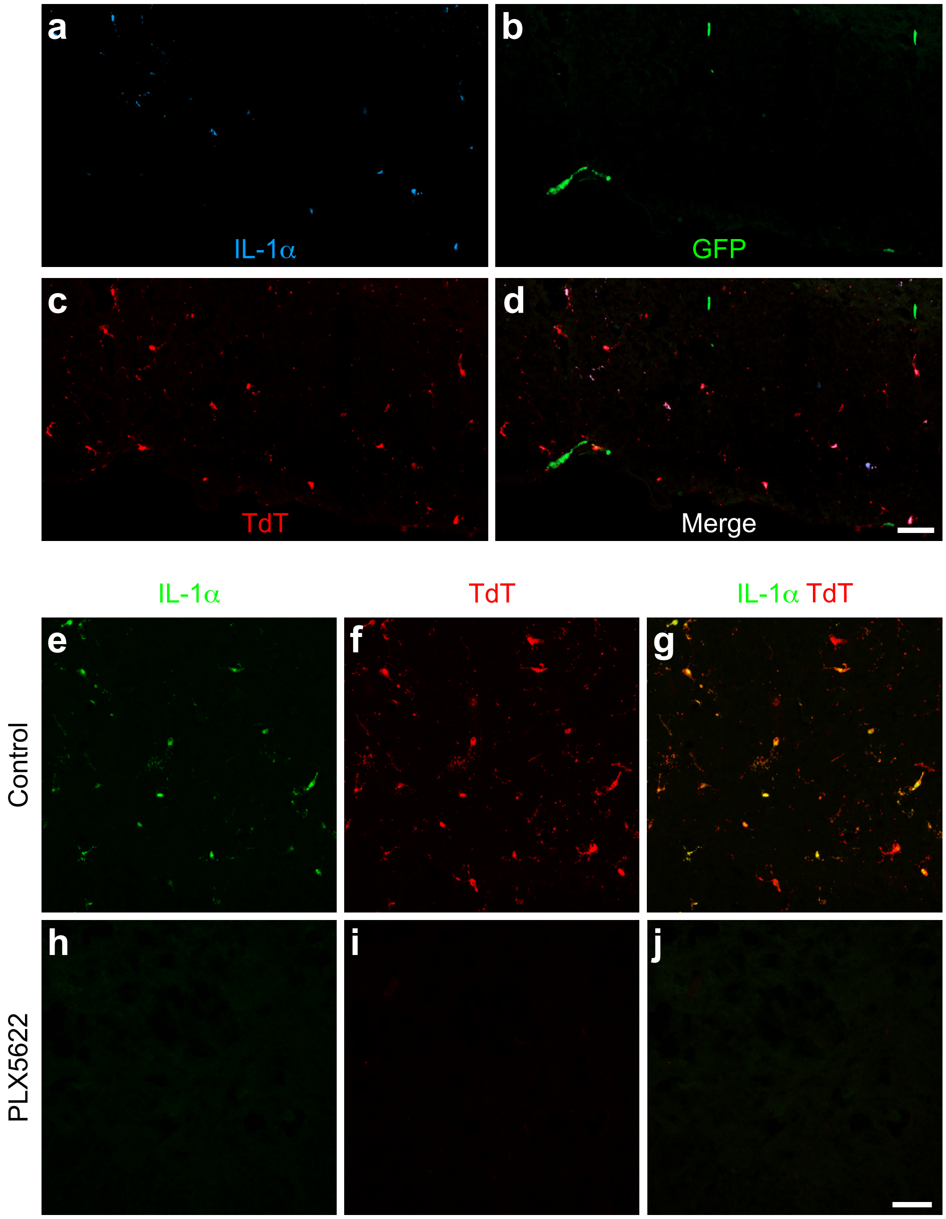

Supplementary Figure 1. Depletion of microglia prior to the time of injury results in the absence of IL-1α staining at sites of SCI. (**A-D**) Representative confocal images showing IL-1α immunostaining (blue, **A**) in the injured spinal cord of a *LysM-eGFP*::Cx3cr1^{CreER}::*R26-TdT* fluorescent reporter mouse, in which blood-derived cells of the granulo-myelomonocytic lineage express GFP (LysM-eGFP, green cells in **B**) and microglia express Td-Tomato (TdT, red cells in **C**), at 4 hours post-SCI. The merged images are shown in **D**. (**E-J**) Representative confocal images showing IL-1α immunostaining (green cells in **E**) in the spinal cord of an injured *Cx3cr1*^{Cre}::*Rosa26*^{TdT} transgenic mouse, in which microglia express the fluorescent reporter TdT (red cells in **F**), at 4 hours post-SCI. Note that immunostaining for IL-1α at the lesion epicenter disappeared in SCI *Cx3cr1*^{Cre}::*Rosa26*^{TdT} mice depleted in microglia with PLX5622 chow (three weeks pretreatment in food pellets, panels **H-J**). Representative images of a single experiment are shown in **A-J**. Scale bar: (**A-D**, in **D**) 50 µm, (**E-J**, in **J**) 50 µm.

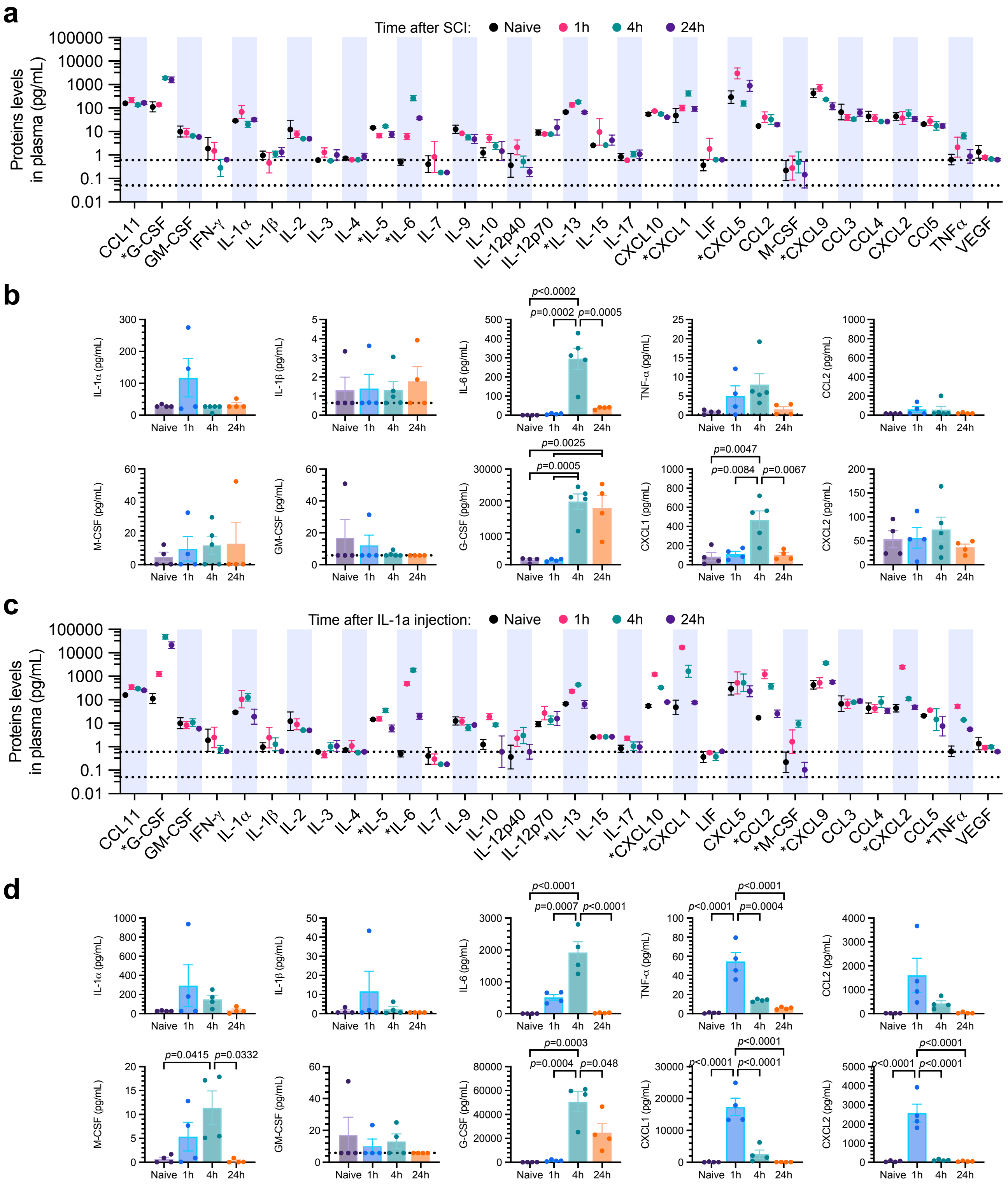

Supplementary Figure 2. Contusion SCI and intra-cisterna magna injection of IL-1α in mice induce a similar profile of cytokines/chemokines in the plasma. (**A-D**) Systemic plasma levels of 32 total cytokines and chemokines were assessed under naive conditions at 3 different time points (1, 4 and 24 hours) post-SCI or IL-1α injection (n=4 mice/group), as measured using a multiplex laser bead assay and the Mouse Cytokine 32-Plex Discovery assay from Eve Technologies. Protein levels are expressed in log of their values in pg/ml (**A , C**), and in pg/ml (**B, D**) for cytokines/chemokines that are significantly upregulated after SCI or IL-1α injection compared to naive conditions. Data are expressed as mean ± SEM. Statistical significance was determined by a two-way ANOVA followed by a Bonferroni post-hoc test. Pairwise comparisons and p-values are indicated in the graphs.

Supplementary Figure 2. Bretheau et al., 2022

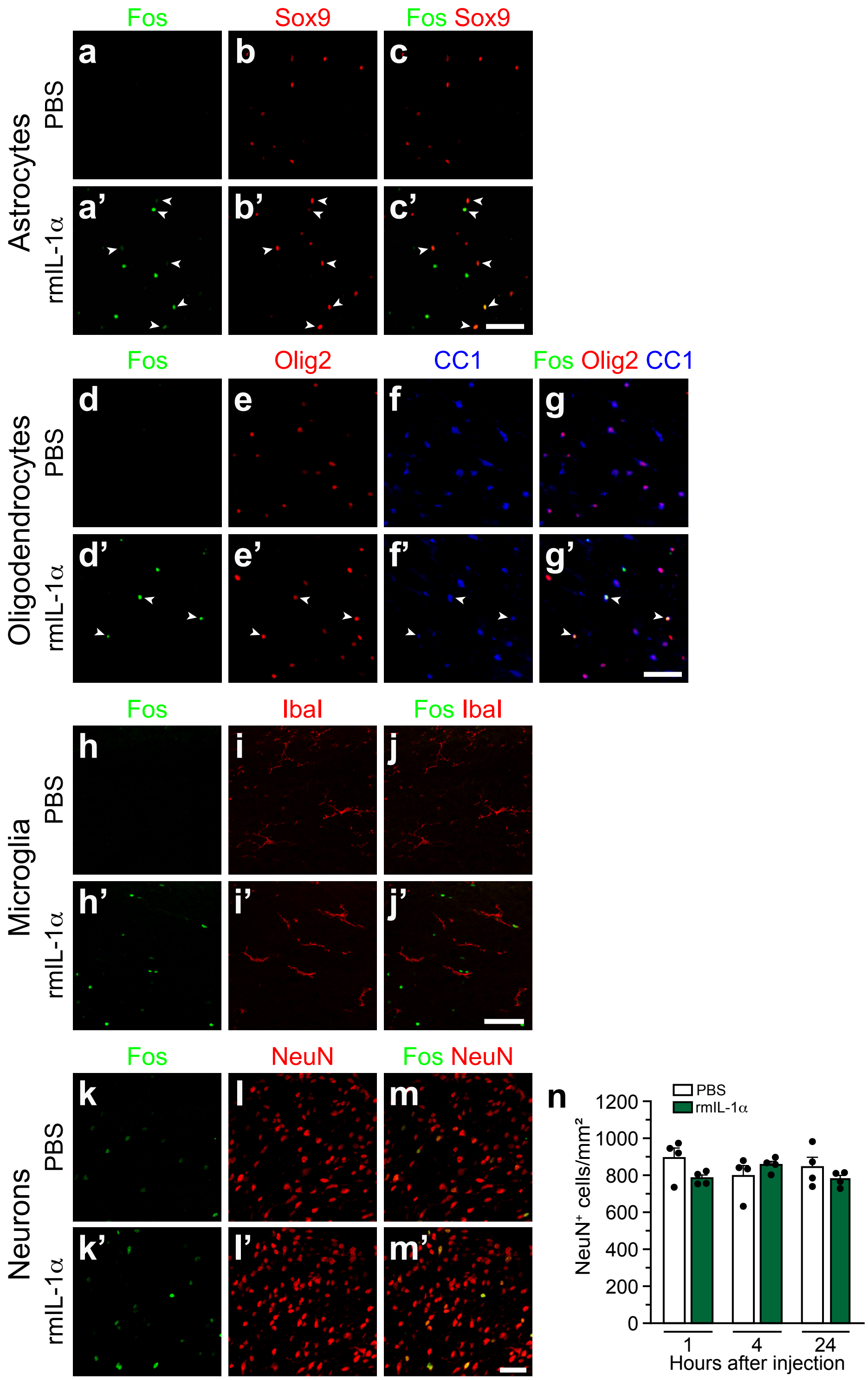

Supplementary Figure 3. Intra-cisterna magna injection of IL-1α in mice induces rapid activation of astrocytes, oligodendrocytes, and neurons the spinal cord. (**A-M'**) Representative confocal images of the spinal cord showing expression of the cell activation marker Fos (green signal) in Sox9$^+$ astrocytes (red cells in **B-C'**), mature Olig2$^+$CC1$^+$ oligodendrocytes (red and blue cells, respectively, in **E-G'**), Iba1$^+$ microglia (red cells in **I-J'**), and NeuN$^+$ neurons (red cells in **L-M'**) at 4 hours after a single i.c.m. injection of either PBS (**A-C**, **D-G**, **H-J**, **K-M**) or rmIL-1α (**A'-C'**, **D'-G'**, **H'-J'**, **K'-M'**). White arrowheads indicate certain double-labeled cells. (**N**) Quantification of the total number of NeuN$^+$ neurons in the spinal cord gray matter of C57BL/6 mice injected with either PBS or rmIL-1α and killed at 1, 4 or 24 hours post-injection (n=4 mice/group). Data are expressed as mean ± SEM. Statistical significance was determined by a two-way ANOVA followed by a Bonferroni post-hoc test. Scale bars: (**A-C'**, in **C'**) 50 µm, (**D-G'**, in **G'**) 50 µm, (**H-J'**, in **J'**) 50 µm, (**K-M'**, in **M'**) 50 µm.

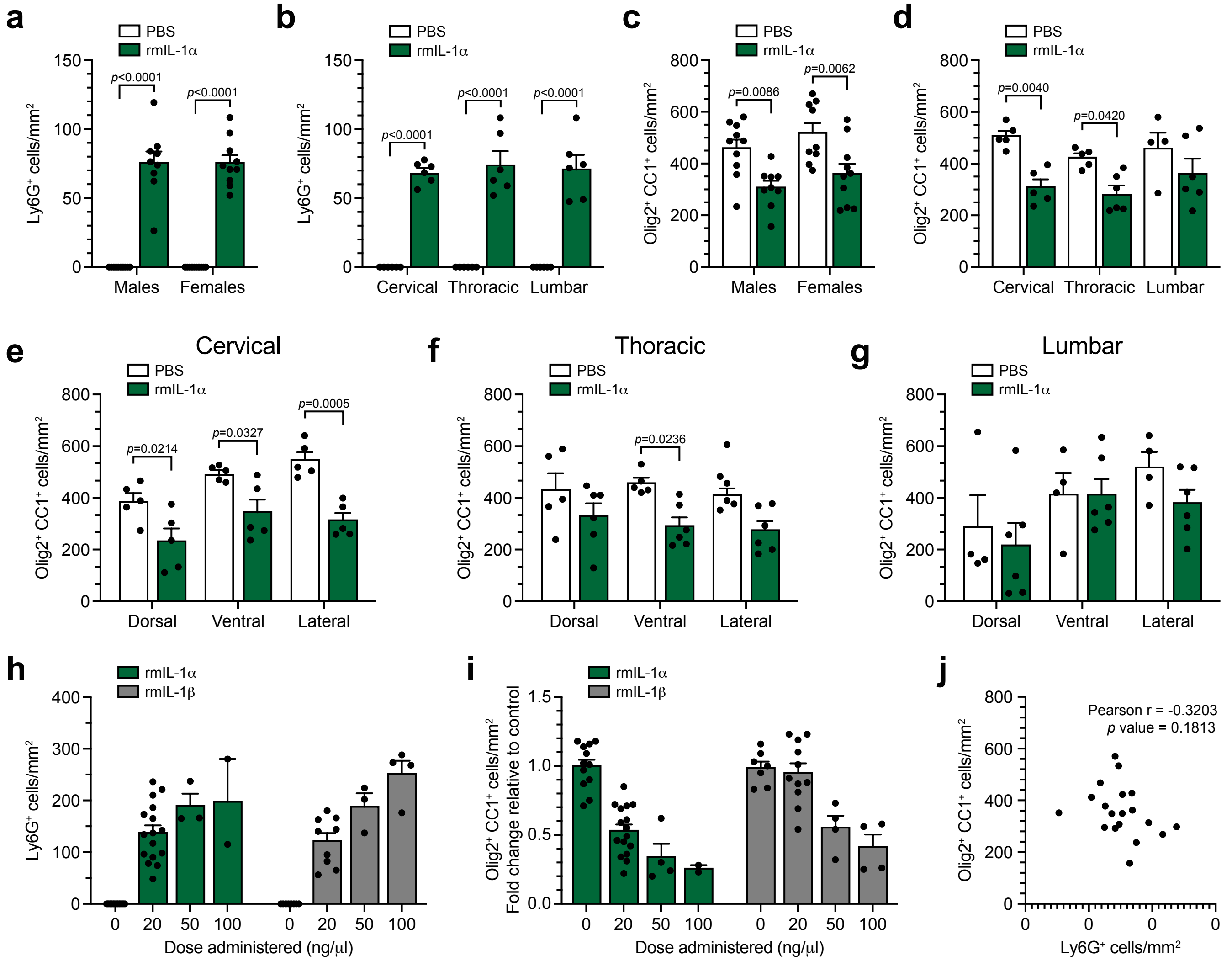

Supplementary Figure 4. IL-1α-mediated neutrophil recruitment and oligodendrocyte loss occur throughout the entire spinal cord independently of biological sex and spinal cord pathways. (**A-B**) Quantification of the total number of Ly6G⁺ neutrophils that infiltrated the spinal cord of male and female C57BL/6 mice (**A**: n=10 Males + PBS, n=9 Males + rmIL-1α, n=10 Females + PBS, n=10 Females + rmIL-1α) as a function of the spinal level (cervical, thoracic, and lumbar spine, in **B**) 24 hours after i.c.m. injection of either PBS or recombinant mouse (rm) IL-1α (**B**: n=6 mice/group). (**C-G**) Quantification of the total number of mature Olig2⁺CC1⁺ oligodendrocytes in the spinal cord of male and female mice (**C**: n=10 Males + PBS, n=9 Males + rmIL-1α, n=9 Females + PBS, n=10 Females + rmIL-1α) as a function of the spinal level (**D**) and white matter pathway (dorsal, ventral, and lateral white matter tracts, in **E-G:** n=4-5 Dorsal + PBS, n=5-6 Dorsal + rmIL-1α, n=4-5 Ventral + PBS, n=5-6 Ventral + rmIL-1α, n=4-6 Lateral + PBS, n=5-6 Lateral + rmIL-1α) at 24 hours post-injection of either PBS or rmIL-1α (n=5-10 mice/group). (**H-I**) Quantification of the total number of infiltrating Ly6G⁺ neutrophils (**H**) and mature Olig2⁺CC1⁺ oligodendrocytes (**I**) in the spinal cord of C57BL/6 mice injected i.c.m. with various doses of IL-1α or IL-1β and killed at 24 hours post-injection (n=12-13 rmIL-1α at 0 ng/µl, n=16 rmIL-1α at 20 ng/µl, n=3-4 rmIL-1α at 50 ng/µl, n=2 rmIL-1α at 100 ng/µl, n=7-8 rmIL-1β at 0 ng/µl, n=9-11 rmIL-1β at 20 ng/µl, n=3-4 rmIL-1β at 50 ng/µl, n=4 rmIL-1β at 100 ng/µl). (**J**) Pearson correlation between the infiltration of neutrophils and OL loss in response to i.c.m. injection of IL-1α. Data are expressed as mean ± SEM. Data are presented as mean values +/- SEM and statistical significance was determined by a two-way ANOVA followed by a Bonferroni post-hoc test, except for **J** where a two-tailed Pearson correlation test was performed. Pairwise comparisons and p-values are indicated in the graphs.

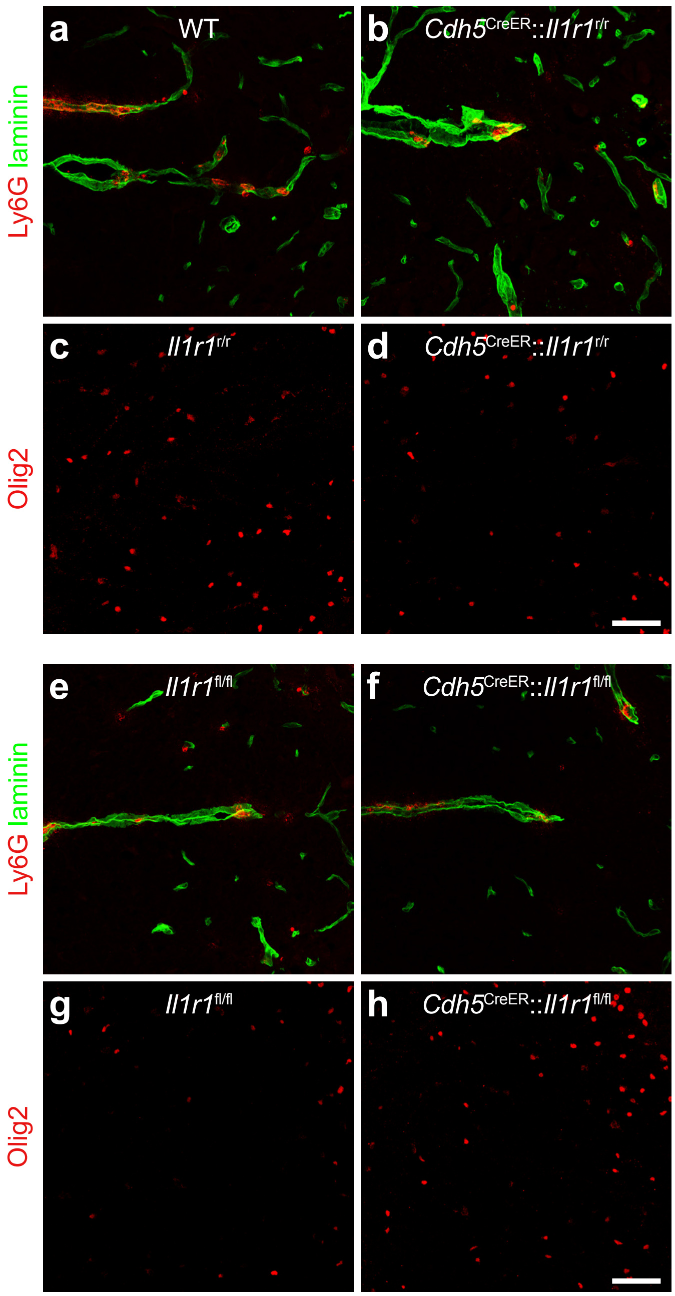

Supplementary Figure 5. Endothelial IL-1R1 partly mediates neutrophil infiltration and oligodendrocyte cell loss after intra-cisterna magna administration of IL-1α. (**A-B**) Representative confocal images showing the infiltration of Ly6G^{+} neutrophils (red cells) in the spinal cord of wild-type (WT) mice (**A**) and *Cdh5*^{CreER}::*Il1r1*$^{\mathrm{r/r}}$ mice (**B**) 24 hours following intra-cisterna magna (i.c.m.) injection with recombinant mouse (rm) IL-1α. An anti-pan-laminin antibody was used to stain blood vessel basement membranes (green staining). (**C-D**) Representative images showing immunostaining for the Olig2 transcription factor, a marker of cells of the oligodendrocyte lineage, in the spinal cord of *Il1r1*$^{\mathrm{r/r}}$ mice (**C**), which express an IL-1R1-knockout phenotype, and *Cdh5*^{CreER}::*Il1r1*$^{\mathrm{r/r}}$ mice (**D**) at 24 hours post-injection of rmIL-1α. (**E-F**) Representative images showing Ly6G^{+} neutrophils (red cells) in the spinal cord of *Il1r1*$^{\mathrm{fl/fl}}$ mice (**E**), which normally express the *Il1r1* gene, and *Cdh5*^{CreER}::*Il1r1*$^{\mathrm{fl/fl}}$ mice (**F**) injected i.c.m. with rmIL-1α and killed at 24 hours post-injection. Laminin immunostaining is shown in green. (**G-H**) Representative confocal images showing immunostaining for Olig2 (red cells) in the spinal cord of *Il1r1*$^{\mathrm{fl/fl}}$ (G) and *Cdh5*^{CreER}::*Il1r1*$^{\mathrm{fl/fl}}$ (**H**) mice at 24 hours post-injection of rmIL-1α. Scale bars: (**A-D**, in **D**) 50 µm, (**E-H**, in **H**) 50 µm.

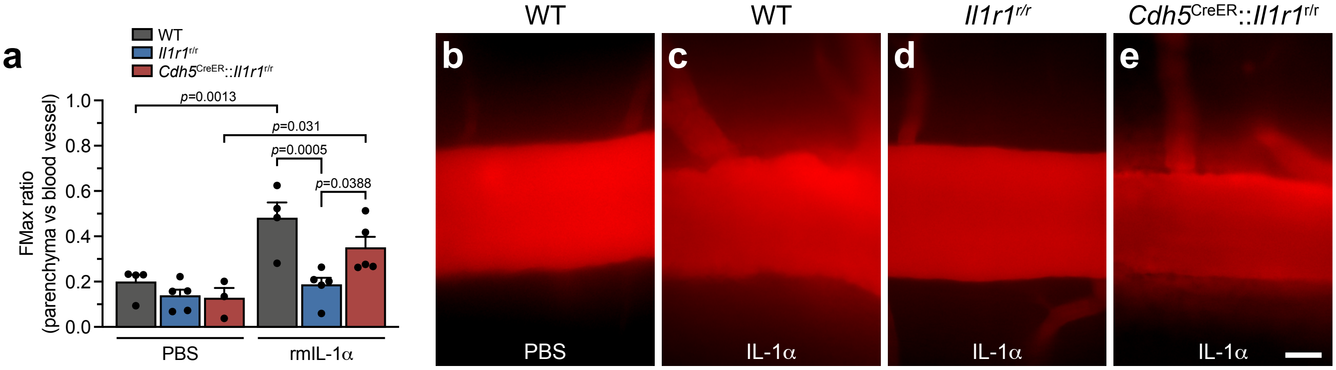

Supplementary Figure 6. Injection of IL-1α into the CNS induces rapid breakdown of the blood-spinal cord barrier through endothelial IL-1R1. (**A**) Quantification of blood-spinal cord barrier (BSCB) leakage in the thoracic spinal cord of adult wild-type (WT), *Il1r1*$^{r/r}$ (which express an IL-1R1-knockout phenotype), and *Cdh5*^{CreER}::*Il1r1*$^{r/r}$ mice at 6 hours post-i.c.m. injection of either PBS or rmIL-1α (n=4 WT + PBS, n=5 Il1r1r/r + PBS, n=3 Cdh5CreER::Il1r1r/r + PBS, n=4 WT + rmIL-1α, n=5 Il1r1r/r + rmIL-1α, n=5 Cdh5CreER::Il1r1r/r + rmIL-1α). The permeability of the BSCB was measured by extravasation of a 40-kDa Texas red-conjugated dextran injected in the tail vein 30 minutes prior to intravital imaging. Data are expressed as maximum fluorescence intensity (Fmax) of Texas Red in the spinal cord parenchyma versus blood vessels and means ± SEM are presented. (**B-E**) Representative intravital fluorescence images captured by videomicroscopy from the spinal cord of mice injected i.v. with the Texas Red fluorescent dye (red) and killed 6 hours after i.c.m. injection of either PBS (**B**) or rmIL-1α (**C-E**). Statistical significance was determined by a two-way ANOVA followed by a Bonferroni post-hoc test. Pairwise comparisons and p-values are indicated in the graphs. Scale bar: (**B-E**, in **E**) 25 µm.

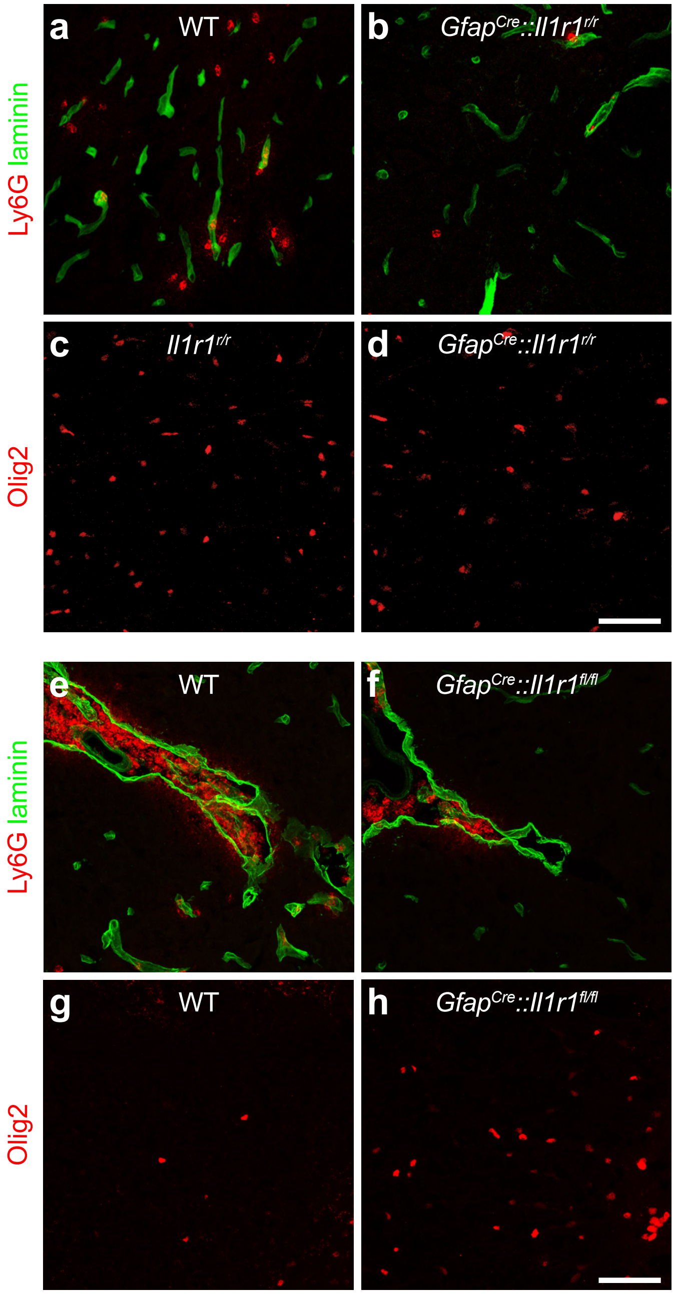

Supplementary Figure 7. Astrocytic IL-1R1 partly mediates oligodendrocyte cell loss after intra-cisterna magna administration of IL-1α. (**A-B**) Confocal images showing Ly6G^{+} neutrophils (red cells) in the spinal cord of WT (**A**) and *Gfap*^{Cre}::*Il1r1*$^{\mathrm{r/r}}$ (**B**) mice injected i.c.m. with rmIL-1α and killed at 24 hours post-injection. An anti-pan-laminin antibody was used to stain blood vessel basement membranes (green). (**C-D**) Images showing immunostaining for the Olig2 transcription factor in the spinal cord of *Il1r1*$^{\mathrm{r/r}}$ mice (**C**), which express an IL-1R1-knockout phenotype, and *Gfap*^{Cre}::*Il1r1*$^{\mathrm{r/r}}$ mice (**D**) at 24 hours post-injection of rmIL-1α. (**E-F**) Images showing Ly6G^{+} neutrophils (red cells) in the spinal cord of WT (**E**) and *Gfap*^{Cre}::*Il1r1*$^{\mathrm{fl/fl}}$ (**F**) mice injected i.c.m. with rmIL-1α and killed at 24 hours post-injection. Laminin immunostaining is shown in green. (**G-H**) Images showing Olig2 immunostaining in the spinal cord of WT (**G**) and *Gfap*^{Cre}::*Il1r1*$^{fl/fl}$ (**H**) mice at 24 hours post-injection of either PBS or rmIL-1α. Representative images of two independent experiments are shown in **A-H**. Scale bars: (**A-D**, in **D**) 50 µm, (**E-H**, in **H**) 50 µm.

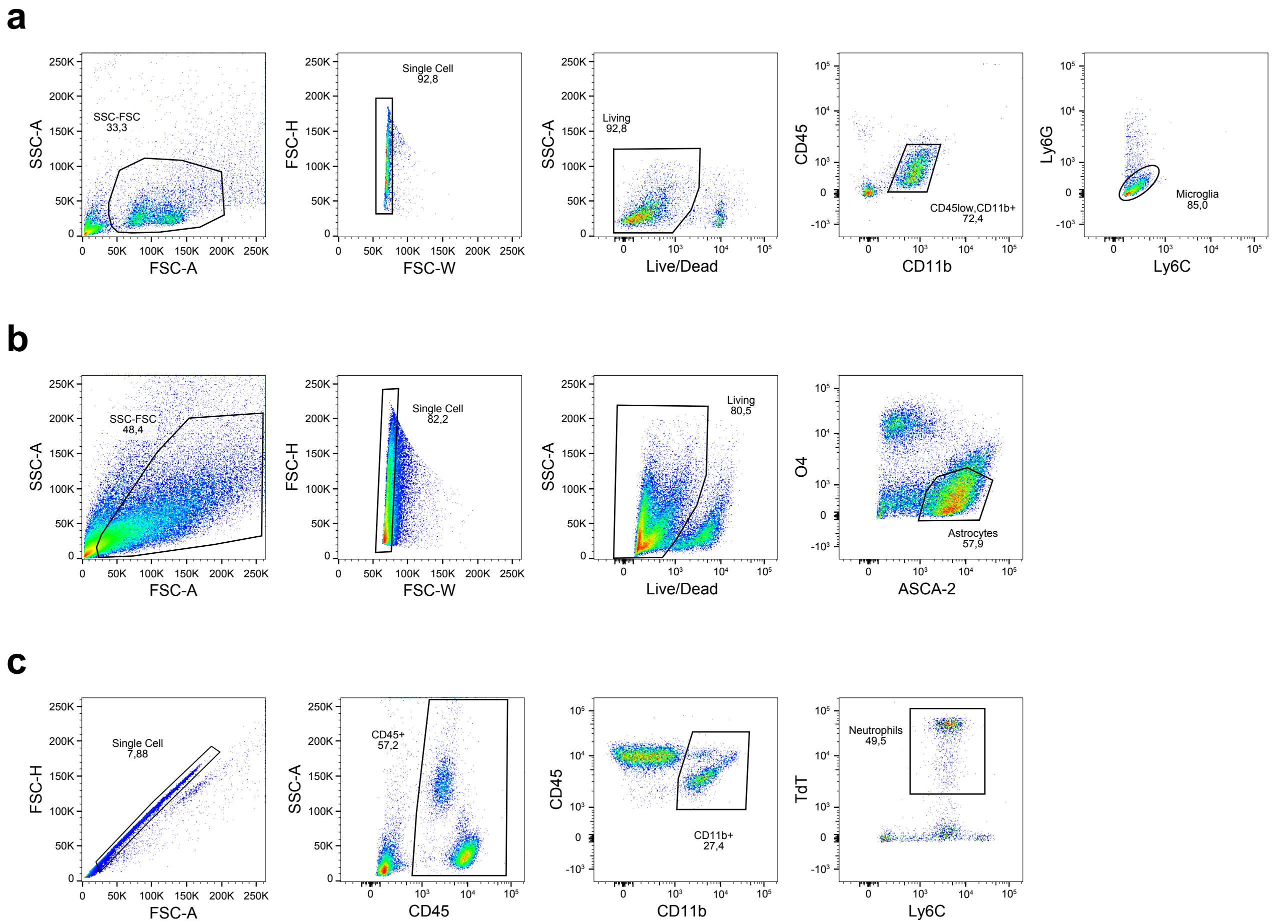

Supplementary Figure 8. Fluorescence-activated cell sorting (FACS) and flow cytometry gating strategies used to identify astrocytes, microglia and neutrophils. (**A**) Gating scheme and representative plots of microglia isolated from the adult spinal cord of *Cx3cr1*^{CreER}::*Il1r1*$^{\text{r/r}}$ and *Cx3cr1*^{CreER}::*Il1r1*$^{\text{fl/fl}}$ mice. (**B**) Gating scheme and representative plots of astrocytes isolated from the adult brain of *Gfap*^{Cre}::*Il1r1*$^{\text{r/r}}$ and *Gfap*^{Cre}::*Il1r1*$^{\text{fl/fl}}$ mice. (**C**) Gating scheme and representative plots of peripheral blood neutrophils isolated from *Ly6g*$^{\text{Cre-TdT}}$ transgenic mice.

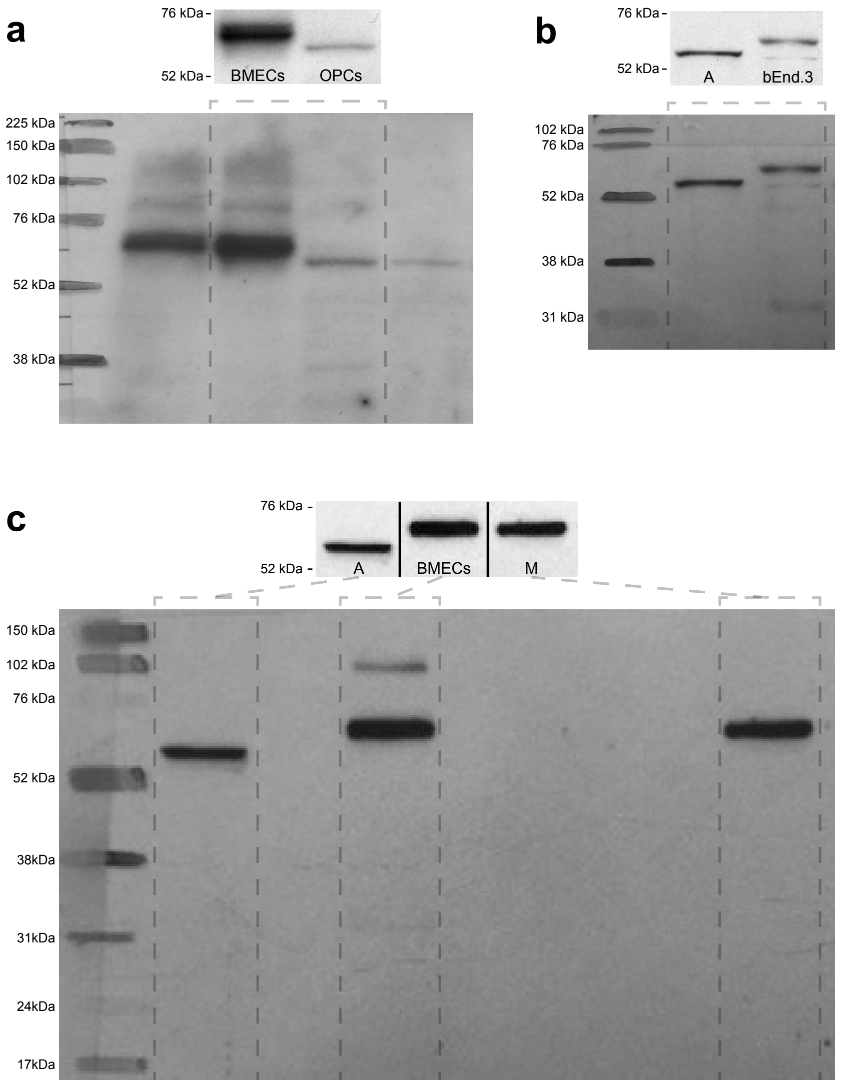

Supplementary Figure 9. Uncropped and unprocessed scans of immunoblots shown in Fig. 4K. Detection by immunoblotting of IL-1R1 in various murine primary and immortalized cells. (**A**) IL-1R1 protein expression in primary brain microvascular endothelial cells (BMECs) and primary oligodendrocyte progenitor cells (OPCs). (**B**) IL-1R1 protein expression in primary astrocytes (A) and immortalized bEnd.3 ECs (bEnd.3). (**C**) IL-1R1 protein expression in primary astrocytes (A), primary BMECs, and primary microglia (M). The dashed boxes indicate the cropped sections shown in the corresponding Fig. 4K. Molecular weight markers (in kDa) are shown on the left.
