# Supplementary material for: The alarmin interleukin-1α triggers secondary degeneration through reactive astrocytes and endothelium after spinal cord injury
Source: Nat Commun. 2022 Oct 2;13:5786. doi: 10.1038/s41467-022-33463-x (PMC9527244; doi:10.1038/s41467-022-33463-x)
Supplement: Supplementary file 3 — Reporting Summary [file 41467_2022_33463_MOESM3_ESM.pdf]

nature portfolio

Corresponding author(s): Dr. Steve Lacroix

Last updated by author(s): Aug 31, 2022

Reporting Summary

Nature Portfolio wishes to improve the reproducibility of the work that we publish. This form provides structure for consistency and transparency in reporting. For further information on Nature Portfolio policies, see our Editorial Policies and the Editorial Policy Checklist.

Statistics

For all statistical analyses, confirm that the following items are present in the figure legend, table legend, main text, or Methods section.

n/a	Confirmed	
☐	☒	The exact sample size (n) for each experimental group/condition, given as a discrete number and unit of measurement
☐	☒	A statement on whether measurements were taken from distinct samples or whether the same sample was measured repeatedly
☐	☒	The statistical test(s) used AND whether they are one- or two-sided<br>*Only common tests should be described solely by name; describe more complex techniques in the Methods section.*
☐	☒	A description of all covariates tested
☐	☒	A description of any assumptions or corrections, such as tests of normality and adjustment for multiple comparisons
☐	☒	A full description of the statistical parameters including central tendency (e.g. means) or other basic estimates (e.g. regression coefficient) AND variation (e.g. standard deviation) or associated estimates of uncertainty (e.g. confidence intervals)
☐	☒	For null hypothesis testing, the test statistic (e.g. F, t, r) with confidence intervals, effect sizes, degrees of freedom and P value noted<br>*Give P values as exact values whenever suitable.*
☒	☐	For Bayesian analysis, information on the choice of priors and Markov chain Monte Carlo settings
☒	☐	For hierarchical and complex designs, identification of the appropriate level for tests and full reporting of outcomes
☐	☒	Estimates of effect sizes (e.g. Cohen's d, Pearson's r), indicating how they were calculated

Our web collection on statistics for biologists contains articles on many of the points above.

Software and code

Policy information about availability of computer code

Data collection	BIOQUANT Life Science software (v. 18.5; Bioquant Image Analysis Corporation), BD FACSDiva software (v. 1.6.3; BD Biosciences), GeneTool software (v. 2.0; BioTools Inc.), Nirvana software (v. 1.9.4; Bliq Photonics), ZEN Blue Edition software (v. 2.3; Carl Zeiss).
Data analysis	FlowJo software (v. 10.8; Tree Star Inc.), GraphPad Prism 9 software (v.9.4.1; GraphPad Software Inc.).

For manuscripts utilizing custom algorithms or software that are central to the research but not yet described in published literature, software must be made available to editors and reviewers. We strongly encourage code deposition in a community repository (e.g. GitHub). See the Nature Portfolio guidelines for submitting code & software for further information.

Data

Policy information about availability of data

All manuscripts must include a data availability statement. This statement should provide the following information, where applicable:

- Accession codes, unique identifiers, or web links for publicly available datasets
- A description of any restrictions on data availability
- For clinical datasets or third party data, please ensure that the statement adheres to our policy

The data that support the findings of this study are available from the corresponding author upon request.

March 2021

Field-specific reporting

Please select the one below that is the best fit for your research. If you are not sure, read the appropriate sections before making your selection.

[x] Life sciences [ ] Behavioural & social sciences [ ] Ecological, evolutionary & environmental sciences

For a reference copy of the document with all sections, see nature.com/documents/nr-reporting-summary-flat.pdf

Life sciences study design

All studies must disclose on these points even when the disclosure is negative.

Sample size	Statistical evaluations were performed using the Student's t-test or one- or two-way ANOVA or repeated-measures ANOVA, as indicated in the figure legends. Post-ANOVA comparisons were made using the Bonferroni correction. For behavioral analyses, the number of mice per group was >8, which represents a >98% probability of detecting a significant change if alpha is set at 0.05 and standard deviations (SDs) are 20% of average. Histological and immunohistochemical comparisons were performed using unpaired Student's t-test (2 groups) or a one-way (>2 groups) ANOVA, followed by post hoc correction. At least 4 mice per condition were used. This represents a >98% probability of detecting a significant change if alpha is set at 0.05 and SDs are 8% of average. Statistical powers were calculated using the maximum SD observed in our previous studies. For the in vitro experiments, the values of 6 wells were averaged for each tested condition.
Data exclusions	No data were excluded from the analyses.
Replication	Data were pooled from two independent experiments for the following figures: Fig. 1A-D, Fig. 6B-C, Fig. 7C-D, Fig. 7I-J, Fig. 8C-D, Fig. 8I-J, and Fig. 10F-L. The results of these experiments were reproducible. Each symbol represents one mouse/well and the total sample size (n) is indicated in the legends.
Randomization	Negative control groups (e.g. genotype, vehicle treatment) were included in all experiments. Male and female mice were randomly assigned (in equal numbers) to control and treatment groups.
Blinding	All quantifications were done blind with respect to the identity of the animals. Behavioral testing was carried out by a blinded investigator.

Reporting for specific materials, systems and methods

We require information from authors about some types of materials, experimental systems and methods used in many studies. Here, indicate whether each material, system or method listed is relevant to your study. If you are not sure if a list item applies to your research, read the appropriate section before selecting a response.

Materials & experimental systems

n/a	Involved in the study	
[ ]	[x]	Antibodies
[ ]	[x]	Eukaryotic cell lines
[x]	[ ]	Palaeontology and archaeology
[ ]	[x]	Animals and other organisms
[x]	[ ]	Human research participants
[x]	[ ]	Clinical data
[x]	[ ]	Dual use research of concern

Methods

n/a	Involved in the study	
[x]	[ ]	ChIP-seq
[ ]	[x]	Flow cytometry
[x]	[ ]	MRI-based neuroimaging

Antibodies

Antibodies used	Neutrophils were depleted through repeated injections of rat anti-mouse Ly6G antibody (50 µg, clone 1A8, BioXCell, BE0075-1) and mouse anti-rat IgG2a (50 µg, clone MAR 18.5, BioXCell, BE0122), while rat IgG2a isotype (50 µg, clone 2A3, BioXCell, BE0089) served as a control.<br><br>Primary antibodies used for immunofluorescence come from the following sources (clone and catalog numbers in parentheses) and were used at the indicated dilutions: rat anti-BrdU (1:1000, clone BU1/75 [ICR1], Abcam, ab6326), rat anti-C3 (1:100, clone 11H9, Abcam, ab11862), mouse anti-CC1 (1:1000, clone CC1, Abcam, ab16794), rat anti-CD11b (1:250, clone 5C6, AbD Serotec, MCA711), rabbit anti-c-Fos (1:500, clone 9F6, Cell signaling, #2250), mouse anti-GalC (1:800, clone mGalC, Millipore, MAB342), goat anti-Iba1 (1:1500, Novus Biologicals, NB100-1028), goat anti-IL-1α (1:100 dilution, R&D Systems, AF-400-NA), rabbit anti-Ki67 (1:200, Abcam, ab15580), rabbit anti-laminin (1:1000, Dako, Z0097), rat anti-Ly6G (1:2000, clone 1A8, BD Biosciences, #551459), mouse anti-NeuN (1:250, clone A60, Millipore, MAB377), rabbit anti-NG2 (1:100, Millipore, Ab5320), rat anti-NG2 (1:200, clone 546930, R&D Systems, MAB6689), mouse anti-O4 (1:400, clone O4, R&D Systems, MAB1326), goat anti-Olig2 (1:400, R&D Systems, AF2418), rabbit anti-P2ry12 (1:500, AnaSpec, AS-55043A), goat anti-Sox9 (1:250, R&D Systems, AF3075), and rabbit anti-Sox9 (1:1000, Millipore, AB5535).<br><br>OPCs and pro-OLs were isolated by immunopanning using a rat anti-PDGFRa/CD140a (1:300, clone APA5, BD Biosciences, #558774) and a mouse anti-O4 (1:300, clone O4, R&D Systems, MAB1326).

Astrocytes were enriched by cell sorting using the APC-conjugated anti-mouse ACSA-2 (1:50, clone REA969, Miltenyi Biotec, 130-116-245) and Alexa 488-conjugated mouse anti-Oligodendrocyte Marker O4 (1:50, R&D Systems, FAB1326G) antibodies.

Antibodies used for flow cytometry are from BD Biosciences (catalog and clone numbers in parentheses) and were used at the indicated dilutions: PerCP-conjugated anti-CD45 (1:50 dilution, clone 30-F11, #557235), Alexa 700-conjugated anti-CD11b (1:50, clone M1/70, #557960), BD HorizonTM V450-conjugated anti-Ly6C (1:83, clone AL-21, #56094), FITC-conjugated anti-Ly6C (1:83, clone AL-21, #553104), and PE-Cy7-conjugated anti-Ly6G (1:50, clone 1A8, #560601).

The following antibodies were used for immunoblotting: mouse anti-actin (1:75000, clone C4, EMD Millipore, MAB1501), rabbit anti-GFAP (1:8000, Agilent Technologies [Dako], Z0334), and goat anti-IL-1R1 (1:1500, R&D Systems, AF-771).

Validation	The antibodies used in this study were chosen based on their extensive use in the literature (supported by the references available on the supplier's website) and a significant amount of experiments in our hands (see the references provided in the Methods section).

Eukaryotic cell lines

Policy information about cell lines

Cell line source(s)	Oligodendrocyte precursor cells (OPCs) were isolated from the neonatal (P7-P9) mouse brain. Endothelial cells were isolated from the brain capillaries of mice aged 6-8 weeks. Mouse primary astrocytes were isolated from the cortex of P0-P2 C57BL/6 mice.
Authentication	Morphology and immunophenotypic characterization were performed to assess the purity of the cultures.
Mycoplasma contamination	The cell lines were not tested for mycoplasma contamination.
Commonly misidentified lines (See ICLAC register)	N/A

Animals and other organisms

Policy information about studies involving animals; ARRIVE guidelines recommended for reporting animal research

Laboratory animals	Male and/or female C57BL/6 mice were purchased from Charles River Laboratories or The Jackson Laboratory (JAX) at 8-10 weeks of age. Cx3cr1CreER mice were obtained from the European Mouse Mutant Archive, with prior authorization from Dr. Steffen Jung (Rehovot, Israel). Breeders for Rosa26-tdTomato (R26-TdT, also known as Ai9, stock #007905), PdgfraCreER (stock #018280), GfapCre (stock #024098), and Il1r1fl/fl (stock #028398) mice were all purchased from JAX. LysM-eGFP knock-in mice were obtained from Dr. Gregory Dekaban (Robarts Institute, London, ON, Canada), with prior consent of Dr. Thomas Graf (Center for Genomic Regulation, Barcelona, Spain). Cdh5CreER mice (line #13073), in which the tamoxifen-inducible Cre recombinase is active in all ECs, were purchased from the Cancer Research Technology Repository at Taconic with prior consent of the creator of the mouse line, Dr. Ralf Adams (London Research Institute, UK). Il1r1r/r mice were obtained from Dr. Ning Quan. Ly6gCre-TdT mice were provided by Dr. Matthias Gunzer (University of Duisburg-Essen, Essen, Germany). All transgenic mouse lines are described in more detail in the manuscript and references provided in it. Mice were housed in individually ventilated cages on racks connected to a central HEPA filtered air supply (30-70 air changes per hour). All animals were kept in a standard 12-h light-dark cycle and had free access to food and water at all times. Room temperature was maintained at 23 ± 2 °C with a relative humidity of 50 ± 5%.
Wild animals	No wild animals were used in the study.
Field-collected samples	No field collected samples were used in the study.
Ethics oversight	All animal procedures were approved by the Comité de protection des animaux de l'Université Laval (protocols #CHU-20-675 and #CHU-21-860) and conducted in compliance with relevant ethical regulations and guidelines of the Canadian Council on Animal Care.

Note that full information on the approval of the study protocol must also be provided in the manuscript.

Flow Cytometry

Plots

Confirm that:

- [x] The axis labels state the marker and fluorochrome used (e.g. CD4-FITC).
- [x] The axis scales are clearly visible. Include numbers along axes only for bottom left plot of group (a 'group' is an analysis of identical markers).
- [x] All plots are contour plots with outliers or pseudocolor plots.
- [x] A numerical value for number of cells or percentage (with statistics) is provided.

Methodology

Sample preparation	AUTOMATED BLOOD CELL COUNT, FLOW CYTOMETRY AND CELL SORTING. Blood was collected via cardiac puncture using a

	22-gauge heparinized syringe. Blood samples were immediately transferred into EDTA-coated microtubes (Sarstedt) and put on slow rotation at 5 rpm (using the Mini LabRollerTM Rotator) until processing was complete. Automated complete blood cell count was performed using the Scil Vet abc Plus+TM Analyzer (Scil Animal Care Company) following manufacturer's instructions. For flow cytometry, red blood cells were lysed and the remaining cells incubated with Mouse Fc Block (i.e., purified anti-mouse CD16/CD32) for 15 minutes at 4°C to prevent nonspecific binding. Multicolor labeling was then performed using the LIVE/DEAD™ Fixable Yellow Dead Cell Stain Kit (Thermo Fisher Scientific) and the following fluorescently-conjugated primary antibodies (all from BD Biosciences): PerCP-conjugated anti-CD45 (1:50 dilution), Alexa 700-conjugated anti-CD11b (1:50), and BD HorizonTM V450-conjugated anti-Ly6C (1:83). Data were acquired on a BD LSRII flow cytometer using the BD FACSDiva software, and further analyzed using the FlowJo software.<br><br>For the purification of microglia required for DNA and mRNA analyses via quantitative real-time PCR (qPCR) and RT-PCR (qRT-PCR), respectively, microglia were isolated from the adult spinal cord as described above and then sorted using a BD FACSAria II. The following primary antibodies were used (all from BD Biosciences): PerCP-conjugated anti-CD45 (1:50 dilution), Alexa 700-conjugated anti-CD11b (1:50), FITC-conjugated anti-Ly6C (1:83), and PE-Cy7-conjugated anti-Ly6G (1:50). Microglia were identified as CD45int CD11b+ Ly6C– Ly6G– cells.
Instrument	FACS LSRII flow cytometer (BD Biosciences).
Software	FACSDiva software (v. 6.1.3, BD Biosciences) was used for data acquisition, and subsequent analysis was performed on FlowJo software (v. 10.8.1, Tree Star Inc.).
Cell population abundance	Myeloid cell populations were analyzed using flow cytometry on blood samples, as described in the "Methods" subsection "Automated blood cell count, flow cytometry and cell sorting". The abundance of each cell population is represented as absolute cell number per mm3 of peripheral blood in Fig. 9B, as calculated based on complete blood cell count results generated using the automated Scil Vet abc Plus+TM veterinary hematology analyzer.
Gating strategy	For fluorescence-activated cell sorting isolation of specific glial cell types, cells were initially gated on a forward scatter area (FSC-A) vs. side scatter area (SSC-A) to select cells, followed by a forward scatter height (FSC-H) vs. forward scatter width (FSC-W) gating to exclude multiplets, and a SSC-A vs. LIVE/DEAD stain (Fixable Yellow Dead Cell Stain Kit) to exclude dead cells. Microglia were sorted using CD45-PerCP low /CD11b-A700 positive and Ly6C-FITC/Ly6G-Pe-Cy7 double negative (CD45int CD11b+ Ly6C– Ly6G– cells). Astrocytes were sorted using ASCA-2-APC and O4-Alexa488 markers.<br><br>For blood neutrophils, doublets were excluded using FSC-A vs. FSC-W gating, total leukocytes filtered with SSC-A/CD45-PerCP and myeloid lineage cells selected with CD11b-A700. Neutrophils were further identified based on TdTomato fluorescence expression.

☒ Tick this box to confirm that a figure exemplifying the gating strategy is provided in the Supplementary Information.
